# The impact of the SKILLZ intervention on sexual and reproductive health empowerment among Zambian adolescent girls and young women: results of a cluster randomized controlled trial

**DOI:** 10.1186/s12978-025-02046-6

**Published:** 2025-06-05

**Authors:** Lila A. Sheira, Chama Mulubwa, Calvin Chiu, Jenala Chipungu, Chelsea Coakley, Helene Smith, Ushma D. Upadhyay, Chansa Chilambe, Besa Chibwe, Jake M. Pry, Boyd Mkandawire, Maggie Musonda, Carolyn Bolton Moore, Jenny Liu

**Affiliations:** 1 Institute for Health & Aging, School of Nursing, University of California, San Francisco, USA; 2 Centre for Infectious Disease Research in Zambia, Lusaka, Zambia; 3 Desmond Tutu HIV Centre, Department of Medicine, University of Cape Town, Cape Town, South Africa; 4 Centre for Social Science Research, University of Cape Town, Cape Town, South Africa; 5 School of Population Health, University of New South Wales, Sydney, Australia; 6 Department of Obstetrics, Gynecology, & Reproductive Sciences, University of California, San Francisco, USA; 7 Division of Epidemiology, School of Medicine, University of California, Davis, USA; 8 Grassroot Soccer Zambia, Lusaka, Zambia

**Keywords:** Adolescent health, Sexual and reproductive health, Empowerment, Unintended pregnancy, Zambia

## Abstract

**Background:**

Zambian adolescent girls and young women (AGYW, age 15–24) experience a disproportionate burden of HIV and unintended pregnancy. Sports-based interventions, which affect sexual health behaviors via improving sexual and reproductive empowerment, may be an innovative and effective approach for promoting HIV and unintended pregnancy prevention. We sought to evaluate the impact of a peer-led, sports-based intervention on sexual and reproductive empowerment among in-school Zambian adolescent girls and young women.

**Methods:**

Data come from the ‘SKILLZ’ study, a cluster randomized controlled trial evaluating the impact of a peer-led, sports-based health education program. Sexual and reproductive empowerment, a secondary outcome of SKILLZ, was measured via the 23-item Sexual and Reproductive Empowerment for adolescents and young adults scale (range 0–92, higher = more sexual and reproductive empowerment) three times over approximately 24 months. We conducted a difference-in-differences analysis to evaluate intervention impact over time.

**Results:**

The study enrolled 2,153 AGYW (1134 intervention; 1019 control) across 46 secondary schools in Lusaka. Median age at baseline was 17; participants were largely unmarried (96%), with 20% reporting any sexual activity. By endline, nearly 40% reported being sexually active. Between baseline and midline, attending an intervention school was associated with a 6.21-point increase in overall score calculated using the imputed sample (standard error [SE]: 0.75, *p* < 0.001) compared to being in a control school (6.75% change). At endline, being in an intervention school was associated with a 5.12-point increase in the Sexual and Reproductive Empowerment overall score (SE: 0.71, *p* < 0.001; 5.57% change)). Among sexually active AGYW, being in an intervention school was associated with a 7.78-point (SE: 1.17, *p* < 0.001) and 4.64-point increase (SE: 0.93, *p* < 0.001) from the baseline to the midline and endline rounds, respectively (8.46% and 5.04% change, respectively).

**Conclusion:**

The intervention moderately impacted Sexual and Reproductive Empowerment scores; results were magnified among sexually active AGYW. Given adolescence is a critical period for sexuality and gender programming, as well as for sexual debut, empowerment interventions at schools may support downstream sexual health behaviors that will impact the life-course of AGYW.

**Trial registration:**

This study was registered in ClinicalTrials.gov (NCT04429061) on March 17th, 2020.

**Supplementary Information:**

The online version contains supplementary material available at 10.1186/s12978-025-02046-6.

## Background

Adolescent girls and young women (AGYW) living in sub-Saharan Africa (SSA) experience pronounced unmet need for contraception [1], directly contributing to high rates of unintended pregnancy [2], unsafe abortions [3], and subsequent poor maternal and pediatric health outcomes [4]. Through similar transmission paths, AGYW in SSA are also vulnerable to HIV acquisition [5, 6], with AGYW accounting for nearly 61% of incident HIV cases in 2022 [7]. Low sexual empowerment [8] and condom negotiating power [9], experiencing barriers in accessing health services [10–12], and living in poverty [13] contribute to and are significant drivers of AGYWs’ risk of unintended pregnancy and HIV acquisition, which have subsequent effects on educational attainment [14, 15], long-term economic well-being [16], morbidity, and mortality [17–21].

There is evidence, predominantly from Western settings, that AGYW participation in sports may be protective against sexual behaviors that increase the risk for pregnancy, HIV, and/or other sexually transmitted infections (STIs) [22–24]. For example, AGYW who engage in sports report higher condom and contraceptive use [25] as well as reduced or lack of engagement in sexual activity compared to those not engaged in sports. While much of this research has focused on team sports, there is evidence that individual sports have protective effects [22]. Further, studies among AGYW globally have demonstrated how engagement in sports programs is associated with increased HIV knowledge and testing [26–29]. Some theorize that the association between participation in sports and sexual behavior may be facilitated through empowerment and self-efficacy [23, 25, 26]. In a study of a sports and empowerment-based program for AGYW at risk of HIV acquisition in South Africa, both empowerment and self-efficacy improved after engagement in sports [26]. Predominantly conducted in sex-segregated settings, this program fostered “safe spaces” to talk in an environment where AGYW could safely and comfortably engage in an activity traditionally dominated or reserved for men (sports) [30].

The connection between engagement in sports and improved sexual and reproductive empowerment (SRE) was empirically tested among 176 women aged 18–19 in the United States, where researchers evaluated whether self-reliance and general self-efficacy mediated the relationship between engaging in sports and behaviors that put the AGYW at risk for pregnancy or STIs [25]. The authors found that, in addition to engaging in sports being protective for sexual health, self-reliance and efficacy mediated both risk behaviors as well as engaging in health-seeking behaviors, such as seeking contraceptives [25]. Experts have posited that this interrelationship was best explained by *cultural resource theory* [24, 25], whereby “cultural scripts” dictating more traditional female behavior are rewritten by engaging in sports, a traditionally male dominated activity [31]. This engagement in sports programs may allow the skills gained in sports, including learning how to be assertive and proactive, to translate to other domains, such as interpersonal relationships. These sports sessions provide AGYW with a testing ground to practice these skills where they might, subsequently, reevaluate gendered norms around sexual empowerment using the same assertive and proactive skills gained [26].

The impact of AGYW-centered sports programming on AGYW SRE as yet to be rigorously evaluated [28], with prior evidence relying on observational data lacking a control group [25], focus group discussions, and other qualitative data [26]. A recent randomized trial evaluating the impact of a sports-based program implemented at schools in South Africa on AGYW pregnancy was unable to be successfully evaluated due in large part to poor attendance in program sessions, precluding meaningful evaluation of its potential [32]. Nevertheless, SRE plays a critical role in the life course of women, including being associated with sexual violence [8, 33], condom [8, 34] and contraceptive use [35], and HIV and STI risk [33]. In turn, interventions which seek to improve SRE during adolescence, a period of significant learning of gender norms [36], may be particularly effective at improving health across women’s life course. To address this research need, this study leverages data from a cluster randomized controlled trial (cRCT) to evaluate the effect of a peer-led, sports-based program on the secondary outcomes of sexual and reproductive health empowerment among school-age Zambian AGYW.

## Methods

### Study setting

This study was conducted in four urban districts of Zambia: Chilanga, Chongwe, Kafue, and Lusaka. The prevalence of HIV is 11% in Zambia, with differences between men (8%) and women (13%), as well as regionally, with the prevalence of HIV in the capital (Lusaka) 14% [37]. There exists a sex disparity, whereby Zambian AGYW aged 20–24 have three times the prevalence of HIV (5.9% vs. 1.8%) as their male peers, and > 10 times the incidence among those 15–24 (1.07% vs. 0.08%) [38], partially explained by the fact that HIV among AGYW is often acquired from older sexual partners [39]. Further, pregnancy among Zambian AGYW is highly prevalent, with 30% of Zambian AGYW having begun childbearing at age 17, and over half at age 19 [40].

### Study design

The ‘SKILLZ’ study (‘parent trial’) is a Type 1 Hybrid Implementation-Effectiveness cRCT [41] evaluating the impact of SKILLZ, a previously piloted [26], peer-led, sports-based program for empowering adolescent girls implemented by Grassroot Soccer (GRS) in Zambia. The primary outcomes of the parent trial were HIV testing and contraceptive use. The effectiveness of this peer-led, sport-based empowerment model on AGYW SRE has not been rigorously evaluated and is a secondary outcome of the trial and focus of this study. Study activities took place in 46 schools from March 2021 to January 2023.

### Participant recruitment and inclusion criteria

Sixty-eight schools in the four selected districts were contacted for screening to determine if they met the eligibility criteria of ≥ 40 AGYW in Grade 11 and had school officials who agreed to participate. Twenty-two schools were excluded due to insufficient eligible AGYW in Grade 11; and 46 schools were recruited and agreed to participate. Schools were stratified by district, location (urban vs. rural), remoteness (quartiles of distance from local District Education Board), size of student body (in quartiles), and school type (co-ed or single sex) and randomized 1:1 to intervention and control arms within strata using guidance from the World Bank’s Development Impact (DIME) guidance on stratification [42].

At each school, 30 eligible AGYW students were randomly pre-selected from school rosters prior to any intervention activities. Eligible AGYW were attending school, at least age 16, in grade 11 at one of the 46 participating schools, and able to provide parental consent as well as individual assent (if under age 18; above 18 consented for themselves). Schools did not provide resourcing nor programming for any of the SKILLZ intervention components.

### Interventions

All AGYW within schools randomized to the SKILLZ intervention were offered four intervention components, independent of their selection to be followed up over the study period:

1. Twelve peer-led after-school sessions commencing after the evaluation baseline survey and which were mostly completed within ~ 6-months of the baseline assessment (see Sars-Cov2 delays below), delivered approximately every 1–2 weeks by trained, young female adult mentors (peer “Coaches” or “near peer mentors” aged 20–30) and lasting 90–120 min, which focused on comprehensive sexual and reproductive health education as well soccer classes;
2. A community “graduation” event within a month (with some variations due to sars-cov2, see Supplementary Fig. 1) of completing all 12 sessions where HIV testing and contraception were available at a mobile clinic staffed by trained nurses/midwives in AGYW-friendly care (weekly topics in Supplementaty Materials 2);
3. Ten follow-on and optional “Teen Club” meetings offered approximately every 1–2 weeks and led by coaches which provided continued opportunities for guided learning about sexual health, goal-planning, empowerment, and well-being, which commenced after the graduation (i.e., took place between midline and endline) and did not include sports programming; and.
4. Community-based distribution of HIV self-tests kits and contraceptives from Coaches trained as community-based distributors during after-school sessions, in addition to referrals to youth-friendly clinic services as needed. While community-based (i.e. not on school premises), these events were open only to AGYW enrolled at SKILLZ schools. The first, second, and fourth components were offered prior to the midline survey wave (with some adjustments due to SARS-CoV-2 related delays, described below); the third component was offered upon conclusion of the after-school sessions and graduation.

Control arm schools implemented the standard of care, which was defined as school-led comprehensive sexual education following the national curriculum [43]. The trial is registered at ClinicalTrials.gov (NCT04429061).

### Impact path from intervention exposure to change in sexual and reproductive health (SRH) empowerment

The after-school sessions’ curriculum focused on empowerment, self-efficacy, SRH, life-skills, body image, and decision-making in relationships, in addition to the sports component. Informed by Social Cognitive Theory [44], this sports and empowerment curriculum model has been previously piloted, with initial evidence for improvements in self-efficacy around sexual behavior and more equitable gender views using single item indicators of these domains (e.g., “A girl’s opinion is as important as a guy’s”), as well as qualitative attestations to feelings of empowerment [26]. Those who participated in at least eight (of twelve) sessions were invited to a graduation ceremony, which we hypothesize served as an empowering event given the community celebration of achieving an outcome in the same school setting where it took place. In regard to the other intervention components’ place on the impact path, we hypothesize that engagement in the community distribution of HIV self-test kits and SRH products component is an *outcome* of increased SRE. Further, the teen club sessions theoretically could impact sexual empowerment at endline, however, they were optional and administrative records from the peers were incomplete, precluding out ability to measure their impact.

### Data collection

Self-administered surveys were given to all participants at baseline, ~ 6- (midline) and ~ 12-months (endline) post baseline (the window for survey rounds extended at the school-level due to SARS-CoV-2 related closures); due to SARS-CoV-2 delays, we refer to encounters with study staff as survey rounds rather than in terms of month. Data were collected via audio-assisted, self-administered surveys conducted at school and on computer tablets with audio narration through headphones in English and two local languages (Bemba and Nyanja). Research staff were nearby to support as needed for clarifications or technical issues. Survey topics included questions on socio-demographic characteristics, sexual history, HIV testing, contraceptive uptake, as well as the complete SRE for Adolescents and Young Adults (SRE for AYA; referred to as SRE) scale.

### SARS-CoV-2 impacts

The study occurred during the global SARS-CoV-2 pandemic in which school closures occurred at different times in Zambia in response to pandemic safety measures. Out of concern for potential increased attrition, halfway through recruitment and enrollment, with institutional review boards (IRB) approval, recruitment targets were increased from 30 to up to 50 AGYW per school. Thus, a second phase of recruiting AGYW occurred, which differentially affected schools given the rolling recruitment across the 46 schools (Supplementary Fig. 1). In some cases, participants received exposure to the intervention prior to receiving an initial assessment; in other words, some AGYW were recruited after the baseline survey wave concluded, and thus were given a first assessment as part of the midline (i.e., 12-month) survey wave. Further, study procedures were paused twice for a three- and one-month period (June 16-August 25, 2021, January 10-February 4, 2022), respectively, during which all intervention and data collections ceased in response to Zambian Government measures to contain the outbreak. As such, 90% of surveys that were scheduled to occur within 6 months of baseline actually occurred within 9 months, introducing variability in the time of completion of the intervention and data collection among participants in both arms. Despite pandemic-related obstacles, the majority of AGYW who were recruited were subsequently enrolled.

### Outcome

We measured SRE via the SRE Scale for Adolescents and Young Adults. The SRE was developed through with the continuous input of adolescents and young people via a rigorous process of qualitative interviews, cognitive testing, literature review, and psychometric analysis [45]. This scale was developed among adolescent populations throughout the United States and captures the diverse experiences and needs of adolescents in their unique life and developmental stage of life, with growing applications in other settings [46–49]. At the time of study roll-out, the SRE was not validated in SSA; it has since been validated in Kenya and used in other countries [46, 47]. The SRE scale has 23 items within seven subscales. Response options include “not at all true” (0), a little true (1), moderately true (2), very true (3), and extremely true (4), summing to a total score range of 0–92 where higher scores are indicative of more SRE [45]. The SRE was administered at each survey round among all participants regardless of engagement in sexual activity as questions are not explicitly related to being ever or currently sexually active; overall scale and subscales showed high internal consistency among the SKILLZ study participants (Table 1).

**Table 1:** Psychometric properties of the SRE scale^1–3^ at baseline among participants missing ≤ 25% of scale items (*n* = 1739), SKILLZ study, Zambia

	*N* Items	Range	Cronbach’s Alpha
Overall SRE score	23	0–92	0.836
Subscales			
Parental support	4	0–16	0.804
Comfort talking with partner	3	0–12	0.696
Choice of partners, marriage, and children	3	0–12	0.657
Sexual safety	4	0–16	0.532
Self-love	4	0–16	0.702
Sense of future	2	0–8	0.590
Sexual pleasure	3	0–12	0.628

^1^Response Options: Not at all true (0), A little true (1), Moderately true (2), Very true (3), Extremely true (4)

^2^Each subscale and overall scored by summing each item

^3^Uses imputed items

### Statistical analysis

Summary statistics of the socio-demographic characteristics of participants at their first survey round are presented, stratified by arm, as well as baseline self-reported proportions of AGYW who have engaged in sexual relations and SRE overall and subscale scores.

To examine the association between exposure to SKILLZ and the SRE scale, we conducted an intent-to-treat analysis and include all participants who were randomly selected from each school, enrolled in the evaluation study, and completed surveys. We conducted a difference-in-difference analysis using a linear model looking at the difference in SRE scores (continuous) by arm at each survey round compared to baseline (i.e. baseline vs. midline, baseline vs. endline). The primary model examined intervention impact from the midline survey round given this is the first follow-up upon completion of the primary components (12 workshops, graduation, and community-based distribution of SRH products) of the intervention. The second model examined sustained intervention impact at 12 months using the same difference-in-difference specifications. Standard errors were clustered within schools, the unit of randomization, and the models controlled for five stratification factors. To facilitate interpretation of effect sizes by arm, the percent change [(Score2-Score1/Total score range [93])*100] is presented for comparability of intervention impact across subscales.

Secondary analyses examined the impact of SKILLZ on the seven sub-domains of the SRE scale using the intent to treat (ITT) analysis and an analysis restricted to those who self-reported being ever sexually active at any of the study survey rounds, using the same model specifications in terms of clustering and controlling for district. For all models, *p*-values adjust for multiple comparisons using a Bonferroni correction [50]. We also conducted a per-protocol analysis, including those in the intervention arm who attended at least eight of twelve sessions as defined in the parent study’s assessment of adherence. Given limitations of the per-protocol analysis [51], we also conducted an instrumental variable analysis, using study arm as the instrument and a binary variable indicating whether the participant attended at least eight of the twelve sessions is the instrumented variable which was interacted with survey round. Results of the instrumental variable analysis thus provide the effect of the “treatment on the treated” [52]. Finally, a fully-adjusted model controlling for month of enrollment (to account for variations/delays in enrollment), variables where baseline imbalance was observed (employment), as well as factors related to the outcome (age and ever sexually active) was conducted. Stata Version 16.1 (College Park, TX) was used for all analyses.

### Approach to missing data

Among participants with ≤ 25% missingness in the SRE items (i.e. ≤6 items), individual items were imputed using multiple imputation and using the responses from the non-missing SRE items in the imputation model as well as the (unimputed) overall score, age, ever being sexually active, mother’s education, and food security status; any missingness in those predictor variables were mean imputed in order to maximize the imputation model. The 25% threshold was chosen as it is more stringent than the “half-rule” which uses a 50% threshold [53], and excessive missingness can lead to imprecise estimation. The imputation was conducted using chained equations which iteratively imputes, allowing items imputed to inform the imputation of other imputed values. At each survey wave, any participant with > 25% missingness of SRE items were excluded.

### Patient and public involvement

There were numerous opportunities for community involvement, including an adolescent community advisory board which included Zambian AGYW in developing and giving feedback on the study tools and processes. Moreover, when the program was first contextualized for Zambia, the peer Coaches were also involved in piloting the content during its first year of implementation.

## Results

**Fig. 1:**
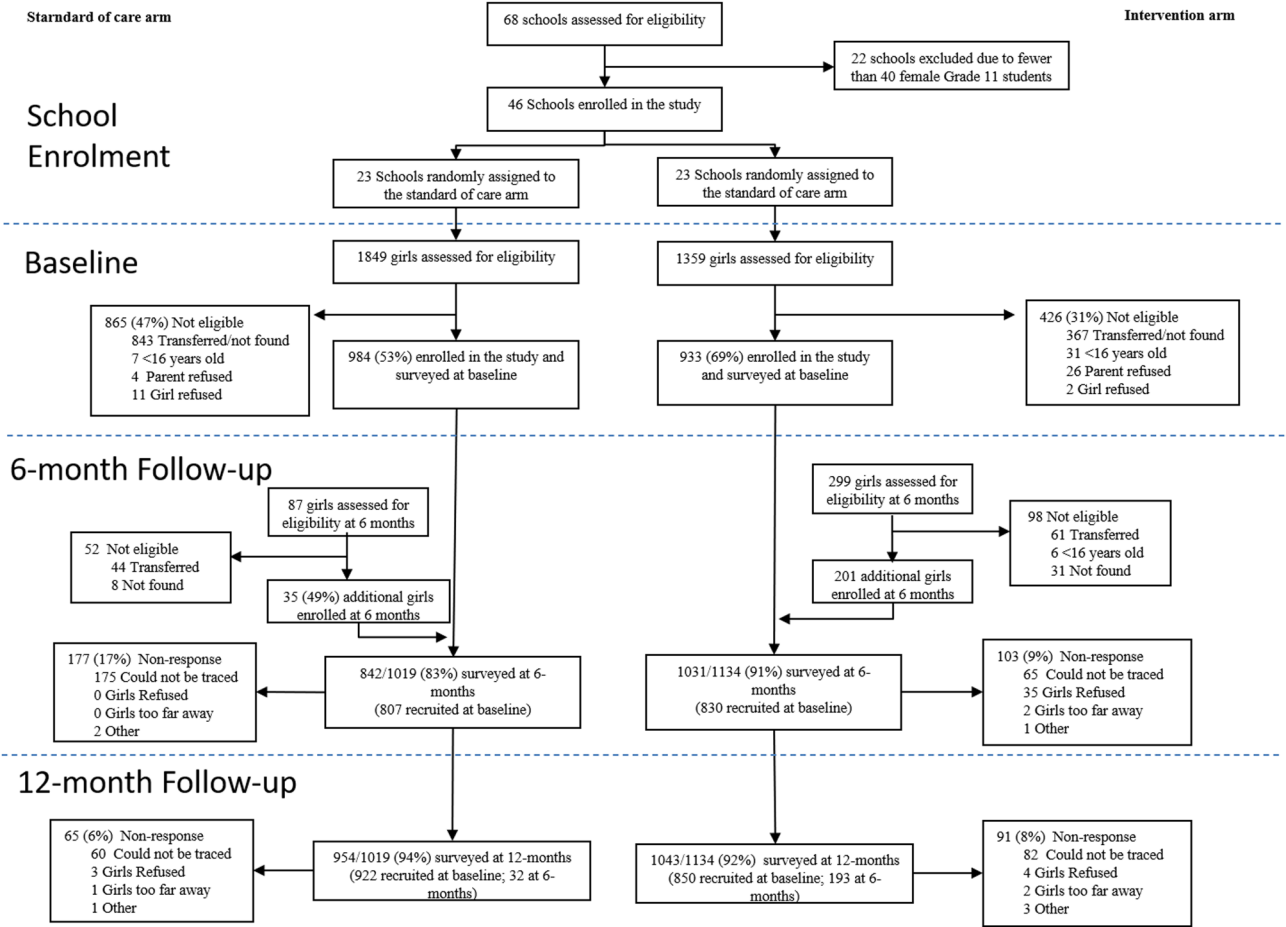
Study CONSORT. Note that there are substantially fewer additionally enrolled participants at the 6-month follow-up in control schools because implementation of the study was staggered across schools. The decision to increase enrolment from 30 to 50 students per school occurred prior to the baseline survey round of 15 out of 23 control schools, but only 9 out of 23 intervention schools, resulting in higher participant recruitment at baseline in more control schools and less need for additional enrolment at the 6-month follow-up

The study assessed 3,208 AGYW for eligibility in Phase 1, of whom 1,917 were eligible and successfully recruited (59.8%). Of those not recruited (*n* = 1,291), 94% (*n* = 1,210) were transferred out/not found to be enrolled at the school during the enrollment period and thus eligibility was not able to be established; 3.3% (*n* = 43) of the 1,291 who were eligible not enrolled were due to refusals (Fig. 1). The remaining 236 (35 control, 201 intervention) were enrolled during Phase 2 after the baseline survey round was completed. Overall, the study enrolled 2,153 AGYW (1019 control, 1134 intervention) from 46 schools across the two enrollment periods (Table 2). The median age of participants at their first survey round was 17 (interquartile range [IQR]: 16, 18). Participants were largely unmarried (95.7%), with nearly one in three (30.6%) experiencing food insecurity in the last 30 days and one in five (19.9%) ever sexually active. Baseline SRE scores were 59.0 and 57.0 in the intervention and control arms, respectively, with similar scores by arm for the subscales (Table 2). The study also experienced high retention, with a combined 87% (*n* = 1,873) and 93% retention (*n* = 1,997) at midline and endline survey rounds (Fig. 1). Engagement in intervention components among participants in the intervention arm was high, with 71% of participating AGYW attending at least eight of twelve sessions.

**Table 2:** Sociodemographic characteristics of study participants at first survey round^1^, SKILLZ Study in Zambia (*n* = 2,153)

	Control (*n* = 1019)	Intervention (*n* = 1134)	Total (*n* = 2153)
Age, median (IQR)	17.0 (16.0, 18.0)	17.0 (16.0, 18.0)	17.0 (16.0, 18.0)
Marital Status			
Unmarried	897 (95.7%)	818 (95.7%)	1715 (95.7%)
Married or divorced	40 (4.3%)	37 (4.3%)	77 (4.3%)
Currently does any job or tasks to earn money	288 (29.3%)	198 (21.3%)	486 (25.4%)
Mother’s educational attainment			
N/A	15 (1.7%)	21 (2.6%)	36 (2.1%)
Primary or less	407 (45.8%)	368 (45.2%)	775 (45.5%)
Some or complete secondary	287 (32.3%)	265 (32.6%)	552 (32.4%)
Post-secondary	179 (20.2%)	160 (19.7%)	339 (19.9%)
Has access to a functional mobile phone?	567 (57.9%)	483 (52.4%)	1050 (55.2%)
Food Insecure	327 (33.5%)	252 (27.5%)	579 (30.6%)
Ever had sexual intercourse	217 (21.8%)	192 (18.0%)	409 (19.9%)
Overall SRE score^2^, median (IQR)	59.0 (50.0, 67.0)	57.0 (48.0, 66.0)	58.0 (49.0, 66.0)
SRE Subscales^3^			
Parental support, median (IQR)	12.0 (10.0, 13.0)	12.0 (10.0, 13.0)	12.0 (10.0, 13.0)
Comfort talking with partner, median (IQR)	6.0 (3.0, 9.0)	6.0 (3.0, 9.0)	6.0 (3.0, 9.0)
Choice of partners, marriage, and children, median (IQR)	9.0 (8.0, 11.0)	9.0 (8.0, 10.0)	9.0 (8.0, 11.0)
Sexual safety, median (IQR)	7.0 (4.0, 10.0)	7.0 (4.0, 10.0)	7.0 (4.0, 10.0)
Self-love, median (IQR)	12.0 (12.0, 16.0)	12.0 (12.0, 15.0)	12.0 (12.0, 15.0)
Sense of future, median (IQR)	6.0 (6.0, 8.0)	6.0 (6.0, 8.0)	6.0 (6.0, 8.0)
Sexual pleasure, median (IQR)	5.0 (2.0, 8.0)	4.5 (2.0, 8.0)	5.0 (2.0, 8.0)
School type			
Co-ed	869 (85.3%)	1083 (95.5%)	1952 (90.7%)
Girls-only	150 (14.7%)	51 (4.5%)	201 (9.3%)
District			
Chilanga	192 (18.8%)	206 (18.2%)	398 (18.5%)
Chongwe	70 (6.9%)	235 (20.7%)	305 (14.2%)
Kafue	260 (25.5%)	94 (8.3%)	354 (16.4%)
Lusaka	497 (48.8%)	599 (52.8%)	1096 (50.9%)
Phase			
Phase A	984 (96.6%)	933 (82.3%)	1917 (89.0%)
Phase B	35 (3.4%)	201 (17.7%)	236 (11.0%)
Sessions Attended out of 12, median (IQR)	--	11 (9, 12)	11 (9, 12)
School in Urban Area (out of 46)	10 (43.5%)	12 (52.2%)	22 (47.8%)
Median N of grade 10 students at school	139 (88, 298)	250 (107, 364)	116 (62, 297)
Median distance in km from district education board	9.5 (6, 30.5)	8 (5, 32)	16 (6, 41)

^1^Responses here include those from original cohort as well as socio-demographic responses from those recruited in the second phase

^2^Range 0-92; higher scores = more sexual and reproductive empowerment

^3^Scores using non-imputed items among those with ≤25% missingness

### Missing data

Participant non-response to SRE items was prevalent; 65% of all participant-waves had complete responses to all 23 items of the SRE score, with 92% of all participant-waves responding to at least 75% of all SRE items. At baseline, midline, and endline survey rounds, 9.3%, 8.2%, and 7.5% of participants had > 25% missingness in the SRE items and were excluded from the analyses. Participants with > 25% missingness at baseline were less likely to have access to a mobile phone, less likely to be sexually active, and were more likely to be from intervention schools compared to the original recruits with ≤ 25% missingness at baseline (Supplementary Table 1). Further, at the midline survey round, 13% (*n* = 280) of participants recruited in combined Phase 1 (*n* = 1917) and Phase 2 (*n* = 236) were not followed up; by endline this was just 7.3% (*n* = 156). Of those with missing visits, they were older, more likely to be food insecure (among those missing the midline survey round), more likely to be sexually active, more likely to be from an intervention school, and had lower overall SRE scores (among those missing the endline survey round) compared to the overall sociodemographic characteristics of those of the original recruits at baseline (Supplementary Table 2).

### Main effect

Between the baseline and midline survey rounds, being in an intervention school was associated with a 6.21-point increase in the SRE overall score calculated (SE: 0.75, *p* < 0.001) compared to being in a control school (Table 3). At endline, being in an intervention school was associated with a 5.12-point increase in the SRE overall score (SE: 0.71, *p* < 0.001) calculated using the imputed items. Enrollment at an intervention school was consistently associated with significantly higher sub-scale scores compared to the control between the baseline survey round and both midline and endline survey rounds (Table 3).

**Table 3:** Difference-in-difference effects of the SKILLZ intervention on SRE overall score and subscales with imputed items among AGYW, SKILLZ Study in Zambia

	Overall sample midline^1^				Overall sample endline^2^			
	D-i-D β	SE	*p*-value^3^	% change	D-i-D β	SE	*p*-value^3^	% change
	Overall population							
Overall SRE score (Range: 0–92) ^4^	6.21	0.75	< 0.001	6.75%	5.12	0.71	< 0.001	5.57%
Subscales ^4^								
Parental support (0–16)	0.63	0.22	0.004	3.71%	0.43	0.2	0.027	2.53%
Comfort talking with partner (0–12)	0.86	0.22	< 0.001	6.62%	0.66	0.17	< 0.001	5.08%
Choice of partners, marriage, and children (0–12)	0.64	0.14	< 0.001	4.92%	0.74	0.16	< 0.001	5.69%
Sexual safety (0–16)	1.33	0.23	< 0.001	7.82%	1.21	0.27	< 0.001	7.12%
Self-love (0–16)	0.82	0.13	< 0.001	4.82%	0.89	0.14	< 0.001	5.24%
Sense of future (0–8)	0.51	0.093	< 0.001	5.67%	0.49	0.077	< 0.001	5.44%
Sexual pleasure (0–12)	1.28	0.21	< 0.001	9.85%	0.66	0.18	< 0.001	5.08%

	Sexually active sample midline ^5^				Sexually active sample endline ^6^			
Overall SRE score (Range: 0–92) ^4^								
Subscales ^4^								
Parental support (0–16)	7.78	1.17	< 0.001	8.46%	4.64	0.93	< 0.001	5.04%
Comfort talking with partner (0–12)								
Choice of partners, marriage, and children (0–12)	0.94	0.34	0.006	5.53%	0.16	0.33	0.64	0.94%
Sexual safety (0–16)	1.04	0.35	0.003	8.00%	0.52	0.25	0.036	4.00%
Self-love (0–16)	0.51	0.21	0.013	3.92%	0.49	0.17	0.003	3.77%
Sense of future (0–8)	1.9	0.26	< 0.001	11.18%	0.89	0.34	0.009	5.24%
Sexual pleasure (0–12)	0.94	0.20	< 0.001	5.53%	1.20	0.19	< 0.001	7.06%
Parental support (0–16)	0.58	0.16	< 0.001	6.44%	0.36	0.12	0.003	4.00%

^1^ 2,050 unique participants comprising 3,459 participant-waves

^2^ 2,053 unique participants comprising 3,586 participant-waves

^3^ Bonferroni adjusted alpha critical value cut off: p<0.00625

^4^Each line a separate regression model; each model adjusted for district, location, remoteness, school type, and number of students

^5^ 830 unique participants comprising 1,399 participant-waves

^6^ 833 unique participants comprising 1,484 participant-waves

### Ever sexually active subset

At the baseline survey round, approximately one in five participants had ever had sex (192 (18%) intervention AGYW; 217 (21.8%) control participants). By the end of the study, 2 in 5 participants reported ever being sexually active (475 (42.1%) in intervention; 389 (38.4%) in control, *p* = 0.08). Between the baseline and midline survey rounds, being in an intervention school was associated with a 7.78-point increase (SE: 1.17, *p* < 0.001) in the SRE overall score calculated, a 25% higher effect compared to the effect which included both ever sexually active AGYW and those who have never been sexually active (Table 3). Between baseline and endline, this increase was 4.64-points (SE: 0.93, *p* < 0.001). With the exception of the *Choice of Partners*,* Marriage*,* and Children* subscale, difference-in-difference estimates across all other sub-scales were larger in magnitude compared to the ITT analysis including both ever sexually active and never sexually active AGYW (Table 3) at the midline survey round; at the endline survey round, estimates were consistently lower compared to the ITT analysis, although still statistically significant. Models examining just the subset of AGYW sexually active at baseline (i.e. not those who sexually debuted during the intervention) are reported in Supplementary Table 3.

### Per protocol

Nearly three out of four (808, 71.3%) intervention participants attended at least eight out of twelve sessions; few (3, 0.33%) attended none. The median number of sessions attended was 11 (IQR: 9, 12). Between baseline and midline, being in an intervention school and receiving sufficient intervention exposure was associated with a 7.56-point increase in the SRE overall score calculated (SE: 0.76, *p* < 0.001) compared to being in a control school (Table 4). Between baseline and endline, the impact was a 6.64-point increase (SE: 0.72, *p* < 0.001).

**Table 4:** Difference-in-difference effects of the SKILLZ intervention on SRE overall score and subscales using per-protocol and instrumental variable analysis, SKILLZ Study in Zambia

	Per Protocol Analysis Midline^1^				Per Protocol Analysis Endline^2^			
	D-i-D β	SE	*p*-value^3^	% change	D-i-D β	SE	*p*-value^3^	% change
	Per-protocol Sample							
Overall SRE score (Range: 0–92) ^4^	7.56	0.76	< 0.001	8.22%	6.64	0.72	< 0.001	7.22%
Subscales ^4^								
Parental support (0–16)	0.82	0.24	0.001	4.82%	0.61	0.19	0.001	3.59%
Comfort talking with partner (0–12)	1.03	0.24	< 0.001	7.92%	0.84	0.19	< 0.001	6.46%
Choice of partners, marriage, and children (0–12)	0.84	0.13	< 0.001	6.46%	0.94	0.15	< 0.001	7.23%
Sexual safety (0–16)	1.66	0.23	< 0.001	9.76%	1.53	0.27	< 0.001	9.00%
Self-love (0–16)	1.02	0.14	< 0.001	6.00%	1.11	0.15	< 0.001	6.53%
Sense of future (0–8)	0.58	0.097	< 0.001	6.44%	0.59	0.08	< 0.001	6.56%
Sexual pleasure (0–12)	1.56	0.20	< 0.001	12.00%	1.02	0.18	< 0.001	7.85%

	**Instrumental Variable Analysis Midline**^**5**^				**Instrumental Variable Analysis Endline**^**5**^			
Overall SRE score (Range: 0–92) ^4^	6.01	0.81	< 0.001	6.53%	5.08	0.84	< 0.001	5.52%
Subscales ^4^								
Parental support (0–16)	1.16	0.23	< 0.001	6.82%	0.8	0.23	0.001	4.71%
Comfort talking with partner (0–12)	0.29	0.25	0.249	2.23%	0.46	0.25	0.067	3.54%
Choice of partners, marriage, and children (0–12)	0.80	0.17	< 0.001	6.15%	0.89	0.17	< 0.001	6.85%
Sexual safety (0–16)	0.68	0.28	0.017	4.00%	0.83	0.29	0.004	4.88%
Self-love (0–16)	1.50	0.18	< 0.001	8.82%	1.35	0.19	< 0.001	7.94%
Sense of future (0–8)	0.89	0.12	< 0.001	9.89%	0.71	0.12	< 0.001	7.89%
Sexual pleasure (0–12)	0.70	0.26	0.007	5.38%	0.037	0.25	0.88	0.28%

^1^ 1,750 unique participants comprising 3,026 participant-waves

^2^ 1,754 unique participants comprising 3,138 participant-waves

^3^ Bonferroni adjusted alpha critical value cut off: *p*<0.00625

^4^Each line a separate regression model; each model adjusted for district, location, remoteness, school type, and number of students

^5^ Instrumental variable analysis leverages all 2,053 participants over two time points (4,306 units)

### Instrumental variable analysis

In the first stage of the instrumental variable analysis, the instrument (treatment assignment) was highly associated with intervention exposure (treatment *p*-value: <0.001; model F-statistic: < 0.001). Receiving sufficient exposure to the intervention was consistently associated with higher SRE scores in the intervention arm compared to baseline and control arms at the midline (6.01 points; SE: 0.81, *p* < 0.001) and endline (5.08 points; SE: 0.84, *p* < 0.001) survey rounds (Table 4). This corresponds to a 6.53% and 5.52% change in score from baseline to midline and endline, respectively. Across subscales (Table 4), scores among those receiving sufficient exposure were in the same direction as the ITT and per-protocol analyses, although slightly attenuated. There were some exceptions, particularly for the *Comfort talking with a partner* subscale, where no significant effect was detected at midline (0.29 points, SE: 0.25, *p* = 0.25) nor endline (0.46 points, SE: 0.25, *p* = 0.067).

### Secondary analyses

In the fully adjusted models, being in an intervention school was associated with a 6.36-point increase in the SRE overall score (SE: 0.76, *p* < 0.001) compared to being in a control school (Table 5). At endline, being in an intervention school was associated with a 5.19-point increase in the SRE overall score (SE: 0.73, *p* < 0.001). When assessing intervention impacts in the sample restricted to just those original recruits, i.e., those who were surveyed prior to intervention start, results were similar in terms of magnitude and direction of effect (results not shown).

**Table 5:** Fully adjusted difference-in-difference effects of the SKILLZ intervention on SRE overall score and subscales, SKILLZ Study in Zambia

	Midline^1^				Endline^1^			
	D-i-D β	SE	*p*-value^2^	% change	D-i-D β	SE	*p*-value^2^	% change
Overall SRE score (Range: 0–92) ^4^	6.36	0.78	< 0.001	6.91%	5.19	0.73	< 0.001	5.64%
Subscales ^4^								
Parental support (0–16)	0.67	0.22	0.002	3.94%	0.43	0.2	0.03	2.69%
Comfort talking with partner (0–12)	0.89	0.22	< 0.001	6.85%	0.65	0.17	< 0.001	5.42%
Choice of partners, marriage, and children (0–12)	0.67	0.14	< 0.001	5.15%	0.76	0.17	< 0.001	6.33%
Sexual safety (0–16)	1.33	0.22	< 0.001	7.82%	1.25	0.27	< 0.001	7.81%
Self-love (0–16)	0.87	0.13	< 0.001	5.12%	0.91	0.14	< 0.001	5.69%
Sense of future (0–8)	0.53	0.093	< 0.001	5.89%	0.5	0.079	< 0.001	6.25%
Sexual pleasure (0–12)	1.27	0.21	< 0.001	9.77%	0.69	0.18	< 0.001	5.75%

^1^2,041 unique participants comprising 3,441 participant-waves

^2^2,046 unique participants comprising 3,572 participant-waves

^3^Bonferroni adjusted alpha critical value cut off: *p* < 0.00625

^4^Each line a separate regression model; each model adjusted for district, location, remoteness, school type, and number of students, month of enrollment, age, employment, and ever sexually active

## Discussion

We demonstrated that a peer-led, sports-based program among in-school Zambian AGYW modestly improved sexual and reproductive health empowerment, an intermediate outcome on the path from the program to increased HIV testing and contraceptive uptake (the primary study outcomes). We found statistically significant changes between baseline and midline in the overall score, as well as on all seven sub-scales, with sustained impacts at endline, 12 months after the primary intervention exposure. The intervention effects were larger in magnitude among ever sexually active participants, and a series of robustness checks accounting for potential threats to internal validity demonstrated consistency in the direction and magnitude of the study’s impact on SRE. This study builds upon observational literature demonstrating the impact of sports programming and engagement in sports on improved sexual health outcomes [22–24], as well as a study which empirically tested whether sexual empowerment mediates that relationship [25]. Our study is the first, to our knowledge, to rigorously evaluate via a cRCT the association of sports programming with SRE, and the first to do so in an African setting.

In terms of size of impact relative to the overall scale range (percent change), the intervention had the greatest measurable effect when using the entire scale and within the subscale domains of comfort talking with a partner, sexual safety, and sexual pleasure. This is likely a direct result of the curriculum, which had explicit sessions focused on health communications, healthy relationships, and how to advocate for oneself, in addition to these themes being consistently referred to throughout other sessions. The intervention did not impact the parental support nor Choice of Partners, Marriage, and Children subscale in terms of percent change compared to other scales. The lack of intervention focus on the sub-domains of parental support stems from parents being seen as an obstacle to promoting sexual well-being and empowerment among AGYW [54, 55]; it is unclear if a focus on these intervention components would have resulted in measurable impacts of greater magnitude that what we observed given it may not have affected parental attitudes directly. While it is unclear what effect size might manifest in behavior change, the intervention did have a measurable impact on the primary outcomes, improving the relative risk (RR) of HIV testing (RR: 1.84, 95% CI: 1.53, 2.15) and contraceptive use (RR: 1.62, 95% CI: 1.24, 2.00) in the intervention compared to the control arm [56].

Among the ever sexually active AGYW, the intervention impact was larger for the overall scale and almost all subscales. Regarding sexual safety and pleasure, the intervention impacts were much larger in terms of magnitude of effect (11% and 14% change in score, respectively) among the ever sexually active participants. Against a backdrop of sexual violence and poverty, where 28% of sexually active participants reported ever experiencing rape and 75% reported ever engaging in transactional sex, there is more potential for these messages to be impactful. Further, the intervention took place during a period of sexual debut; compared to one in five AGYW ever being sexually active at baseline, by endline, over one in three (36.6%, non-differential by arm) reported ever being sexually active. This is consistent with multiple studies reporting the average age of sexual debut in SSA to be around ages 15–16 [57] and with most AGYW being sexually active by age 18 [58]. In Zambia, the Violence against Children and Youth Surveys (VACS) found 27.6% of AGYW had first sex ≤ 15 years of age [59]. There have been conflicting effects of interventions at schools on delaying sexual debut in low- and middle-income countries, with both null and significant effects reported [60, 61]; such interventions have low utility in reducing non-consensual sex. The SKILLZ intervention did not explicitly promote abstinence in line with global calls for girl-friendly, comprehensive sexual education [62]. While control schools offered comprehensive sexuality education per Zambian guidelines, this has been demonstrated to be subject to teacher discretion and limited prioritization among those tasked with leading the curriculum [63, 64]. Given early sexual debut, often due to non-consensual sex, gender disempowerment, and masculine norms [65, 66], is a significant risk factor for HIV acquisition [67], the timing of the intervention to overlap during a period of adolescence when AGYW are becoming sexually active is a promising approach.

Non-sports behavioral interventions which seek to address sexual empowerment have focused on parallel domains of gender and power relations. A 2020 systematic review of SRH interventions for young people in lower- and middle-income countries reported that of the 55 studies included, 17 had significant effects on the various SRH outcomes, including STI knowledge, HIV testing, attitudes around contraceptives, and condom negotiation self-efficacy [68]. The review revealed characteristics of these effective intervention designs including SRH, STI, gender equity education, in addition to psychosocial components such as problem-solving, communication, and assertiveness, all components interwoven into the SKILLZ curriculum. Nevertheless, a similar evaluation of SKILLZ in 38 South African schools found null results on the primary outcomes of HIV, pregnancy, and HIV testing, despite parallel curricula, and that the intervention was also not associated with improved gender roles and self-efficacy and agency, with some indication of impact among those who attended at least seven sessions [29, 32]. The investigators further reported that safety concerns for getting home after the sessions, as well as competing demands on time afterschool may have contributed to the poor attendance, with 27.8% graduating from the program [29]; in our study in Zambia, 89.6% graduated from the program. Similarly, a recent cRCT evaluating the impact of a two-year AGYW-focused empowerment program (with no sports components) with weekly, peer-led “meetings” in the community reported null effects on gender equity and IPV acceptability. Similar to the SKILLZ intervention in South Africa, the authors reported low participant engagement, with 30% of the study participants participating in at least half of sessions, and similar demands on participants’ time [69]. The juxtaposition of these studies highlight that the success of SKILLZ in Zambia is likely attributable to high participant engagement which was further enabled by fewer competing demands outside of school, the intervention taking place at school, and a curriculum in concordance with numerous evidence-based SRH themes.

This study had substantial strengths. It boasted a large sample size of over 2,000 AGYW across 46 schools and three time points. Further, despite global shutdowns related to SARS-CoV-2, there was 94% retention or participation over the study period, high even in the absence of a global pandemic. The cRCT design makes this study among the first to evaluate a sports-based SRH program with a control group, facilitating a more rigorous evaluation of sports SRH programming compared to prior observational studies, as well as the sustained impact of the intervention at approximately one year post baseline, approximately six months after most intervention activities had concluded. Nevertheless, findings must be interpreted within the context of its limitations. This study took place during the global pandemic, resulting in recruitment and the implementation delays. Sensitivity analyses which evaluated the potential bias of this limitation support the hypothesis that this had minimal impact on the measure of the intervention’s impact. There are also limitations relation to our measure of SRE. The SRE scale utilized in this study was not validated in SSA at time of survey, but has since been adapted in Kenya with attention to new item creation for the specific East African context, item revision, and linguistic concerns for use in the East African context [47]. Nevertheless, better than single item proxies, the SRE scale showed high internal validity on the overall and subscales, comparable to original validation study [45]. Our study data had prevalent missingness, which was not restricted to the SRE scale, whereby nearly one in three SRE scales had some degree of missingness, and with the missingness being more prevalent among sexually inactive AGYW, those without access to a phone, and those in intervention schools. Given potential implications for statistical power, biased estimates, and invalid conclusions and to address this potential threat to validity, we utilized multiple imputation, as well as a series of robustness checks. This missingness may be due to the self-administered nature of the survey; this choice was made due in response to the well-documented impact of social desirability bias on reporting of sexual behavior [70].

### Conclusion and future directions

The SKILLZ intervention was effective at improving SRE among in-school Zambian AGYW across four districts of Lusaka. The intervention’s success was bolstered by its high attendance, whereby ~ 90% of AGYW randomly selected for follow up participated in at least eight of twelve offered sessions. The goal of this research was to show that an integrated approach to reaching adolescent, in-school AGYW with critical sexual health and sports-based programming is feasible, acceptable, and can improve outcomes among AGYW during a critical development period. Future adaptations in other settings could also measure the impact of the intervention on physical and mental health, given their pertinence to AGYW well-being. This model may be generalizable to AGYW coming to the age of sexual debut in high HIV burdened settings in other parts of Zambia and outside, and in turn, may promote early sexual empowerment and subsequent increases in sexual healthcare seeking behaviors.

## Supplementary Information

Supplementary Material 1.

## Data Availability

Data is available from the principal investigators upon rasonable request and with a submitted data request form. The full study protocol is available from the principal investigators upon request.

## Abbreviations

AGYW: Adolescent girl and young women
SRE: Sexual and reproductive empowerment
SSA: Sub-Saharan Africa
STI: Sexually transmitted diseases
GRS: Grassroot Soccer
AYA: Adolescents and young adults
