# Supplementary material for: The impact of the SKILLZ intervention on sexual and reproductive health empowerment among Zambian adolescent girls and young women: results of a cluster randomized controlled trial
Source: Reprod Health. 2025 Jun 5;22:95. doi: 10.1186/s12978-025-02046-6 (PMC12142864; doi:10.1186/s12978-025-02046-6)
Supplement: Supplementary file 1 — Supplementary Material 1. [file 12978_2025_2046_MOESM1_ESM.zip › Supplementary Materials.docx]

**Supplementary Materials**

| **Supplementary Table 1: Sociodemographic characteristics of original study recruits at first survey round by missing status, SKILLZ Study in Zambia** | | | | |
| --- | --- | --- | --- | --- |
|  | Complete Case^1^ | Missing ≤25% | Missing>25% | Total |
|  | (n=1202) | (n=1739) | (n=178) | (n=1917) |
| **Age, median (IQR)** | 17.0 (16.0, 18.0) | 17.0 (16.0, 18.0) | 17.0 (16.0, 18.0) | 17.0 (16.0, 18.0) |
| **Marital Status** |  |  |  |  |
| Unmarried | 1098 (95.6%) | 1566 (95.5%) | 149 (98.0%) | 1715 (95.7%) |
| Married or divorced | 51 (4.4%) | 74 (4.5%) | 3 (2.0%) | 77 (4.3%) |
| **Currently does any job or tasks to earn money** | 311 (25.9%) | 440 (25.3%) | 46 (26.0%) | 486 (25.4%) |
| **Mother’s educational attainment** | |  |  |  |
| N/A | 21 (1.9%) | 33 (2.1%) | 3 (2.2%) | 36 (2.1%) |
| Primary or less | 499 (45.0%) | 707 (45.1%) | 68 (50.4%) | 775 (45.5%) |
| Some or complete secondary | 361 (32.6%) | 513 (32.7%) | 39 (28.9%) | 552 (32.4%) |
| Post-secondary | 227 (20.5%) | 314 (20.0%) | 25 (18.5%) | 339 (19.9%) |
| **Has access to a functional mobile phone?** | 695 (57.9%) | 984 (56.9%) | 66 (38.2%)^3^ | 1050 (55.2%) |
| **Food Insecure** | 374 (31.3%) | 523 (30.4%) | 56 (32.6%) | 579 (30.6%) |
| **Ever had sexual intercourse** | 297 (24.8%) | 391 (22.7%)^2^ | 18 (5.4%)^3^ | 409 (21.6%) |
| **Overall SRE score, median (IQR)** | 60.0 (51.0, 68.0) | 58.0 (49.0, 66.0) | -- | 58.0 (49.0, 66.0) |
| Parental support, median (IQR) | 12.0 (10.0, 13.0) | 12.0 (10.0, 13.0) | -- | 12.0 (10.0, 13.0) |
| Comfort talking with partner, median (IQR) | 7.0 (4.0, 9.0) | 6.0 (3.0, 9.0) | -- | 6.0 (3.0, 9.0) |
| Choice of partners, marriage, and children, median (IQR) | 9.0 (9.0, 11.0) | 9.0 (8.0, 11.0) | -- | 9.0 (8.0, 11.0) |
| Sexual safety, median (IQR) | 8.0 (5.0, 11.0) | 7.0 (4.0, 10.0) | -- | 7.0 (4.0, 10.0) |
| Self-love, median (IQR) | 12.0 (12.0, 15.0) | 12.0 (12.0, 15.0) | -- | 12.0 (12.0, 15.0) |
| Sense of future, median (IQR) | 6.0 (6.0, 8.0) | 6.0 (6.0, 8.0) | -- | 6.0 (6.0, 8.0) |
| Sexual pleasure, median (IQR) | 6.0 (3.0, 9.0) | 5.0 (2.0, 8.0) | -- | 5.0 (2.0, 8.0) |
| **Arm** |  |  |  |  |
| Control | 668 (55.6%) | 911 (52.4%) | 73 (41.0%) | 984 (51.3%) |
| Intervention | 534 (44.4%) | 828 (47.6%)^2^ | 105 (59.0%)^3^ | 933 (48.7%) |
| **School type** |  |  |  |  |
| Co-ed | 1085 (90.3%) | 1576 (90.6%) | 160 (89.9%) | 1736 (90.6%) |
| Girls-only | 117 (9.7%) | 163 (9.4%) | 18 (10.1%) | 181 (9.4%) |
| **District** |  |  |  |  |
| Chilanga | 236 (19.6%) | 330 (19.0%) | 25 (14.0%) | 355 (18.5%) |
| Chongwe | 151 (12.6%) | 212 (12.2%) | 28 (15.7%) | 240 (12.5%) |
| Kafue | 181 (15.1%) | 263 (15.1%) | 31 (17.4%) | 294 (15.3%) |
| Lusaka | 634 (52.7%) | 934 (53.7%) | 94 (52.8%) | 1028 (53.6%) |

^1^Among those with 100% completion of SRE

^2^ Indicates a statistically significant difference compared to the 0% missing sample (n=1202) using a chi-squared test of independence

^3^ Indicates a statistically significant difference compared to the ≤25% sample (n=1739) using a chi-squared test of independence

| **Supplementary Table 2: Sociodemographic characteristics at first survey round among those with missing visits^1^, SKILLZ Study in Zambia** | | | |
| --- | --- | --- | --- |
|  | Missing midline visit | Missing endline visit | Total^1^ |
|  | (n=280) | (n=156) | (n=1917) |
| **Age, median (IQR)** | 18 (17, 18)^2^ | 17.5 (16.5, 18.5)^2^ | 17.0 (16.0, 18.0) |
| **Marital Status** |  |  |  |
| Unmarried | 248 (93.6%) | 129 (94.9%) | 1715 (95.7%) |
| Married or divorced | 17 (6.4%) | 7 (5.2%) | 77 (4.3%) |
| **Currently does any job or tasks to earn money** | 81 (29.0%) | 40 (27.6%) | 486 (25.4%) |
| **Mother’s educational attainment** | |  |  |
| N/A | 5 (2.0%) | 3 (2.3%) | 36 (2.1%) |
| Primary or less | 118 (47.0%) | 66 (50.0%) | 775 (45.5%) |
| Some or complete secondary | 84 (33.5%) | 35 (26.5%) | 552 (32.4%) |
| Post-secondary | 44 (17.5%) | 28 (21.2%) | 339 (19.9%) |
| **Has access to a functional mobile phone?** | 166 (59.9%) | 75 (52.1%) | 1050 (55.2%) |
| **Food Insecure** | 1104 (38.0)^3^ | 48 (33.8%) | 579 (30.6%) |
| **Ever had sexual intercourse** | 114 (41.8%)^3^ | 47 (32.9%)^3^ | 409 (21.6%) |
| **Overall SRE score, median (IQR)** | 55.0 (45.5, 65) | 53.0 (42.0, 64.0)^2^ | 58.0 (49.0, 66.0) |
| Parental support, median (IQR) | 12.0 (10.0, 13.0)^2^ | 12.0 (10.0, 13.0) | 12.0 (10.0, 13.0) |
| Comfort talking with partner, median (IQR) | 7.0 (3.0, 9.0) | 6.0 (3.0, 9.0) | 6.0 (3.0, 9.0) |
| Choice of partners, marriage, and children, median (IQR) | 9.0 (7.0, 10.0)^2^ | 9.0 (8.0, 10.0) | 9.0 (8.0, 11.0) |
| Sexual safety, median (IQR) | 7.0 (4.0, 10.0) | 7.5 (4.5, 11.0) | 7.0 (4.0, 10.0) |
| Self-love, median (IQR) | 12.0 (12.0, 14.0)^2^ | 12.0 (12.0, 14.5) | 12.0 (12.0, 15.0) |
| Sense of future, median (IQR) | 6.0 (6.0, 8.0)^2^ | 6.0 (6.0, 8.0) | 6.0 (6.0, 8.0) |
| Sexual pleasure, median (IQR) | 5.0 (3.0, 8.0) | 5.0 (3.0, 8.0) | 5.0 (2.0, 8.0) |
| **Arm** |  |  |  |
| Control | 177 (63.2%) | 65 (41.7%) | 984 (51.3%) |
| Intervention | 103 (36.8%)^3^ | 91 (58.3%)^3^ | 933 (48.7%) |
| **School type** |  |  |  |
| Co-ed | 2339 (85.4%) | 144 (92.3%) | 1736 (90.6%) |
| Girls-only | 41 (14.6%)^3^ | 12 (7.7%) | 181 (9.4%) |
| **District** |  |  |  |
| Chilanga | 44 (15.7%) | 22 (14.1%) | 355 (18.5%) |
| Chongwe | 40 (14.3%) | 20 (12.8%) | 240 (12.5%) |
| Kafue | 49 (17.5%) | 35 (22.4%) | 294 (15.3%) |
| Lusaka | 147 (52.5%) | 79 (50.6%) | 1028 (53.6%) |

**Supplementary Figure 1: Timeline of Data Collection and Implementation of SKILLZ at each school**

| **Year** | | **2021** | | | | | | | | | | **2022** | | | | | | | | | | | |
| --- | --- | --- | --- | --- | --- | --- | --- | --- | --- | --- | --- | --- | --- | --- | --- | --- | --- | --- | --- | --- | --- | --- | --- |
| **Month** | | Mar | Apr | May | Jun | Jul | Aug | Sep | Oct | Nov | Dec | Jan | Feb | Mar | Apr | May | Jun | Jul | Aug | Sep | Oct | Nov | Dec |
| **COVID-19 Disruptions** | |  |  |  |  | | |  |  |  |  |  | |  |  |  |  |  |  |  |  |  |  |
| **Phase** | **District** |  |  |  |  |  |  |  |  |  |  |  |  |  |  |  |  |  |  |  |  |  |  |
| 1 | CHONGWE |  |  |  |  |  |  |  |  |  | | | |  |  |  |  | |  |  |  |  |  |
| 1 | CHONGWE |  |  | |  |  |  |  |  |  | | | |  |  |  |  |  | | | | |  |
| 1 | CHONGWE |  |  | |  |  |  |  |  |  | | | |  |  |  |  | | | | | |  |
| 2 | CHONGWE |  |  |  |  |  |  |  |  | |  |  |  |  | | | |  |  |  | | | |
| 2 | CHONGWE |  |  |  |  |  |  |  |  | | |  |  |  |  |  | |  |  |  |  | | |
| 1 | KAFUE |  |  | |  |  |  |  | |  | | | |  |  |  |  | | | | | |  |
| 1 | KAFUE |  |  | |  |  |  |  | |  |  |  |  |  |  |  |  |  | | | | |  |
| 1 | CHILANGA |  | |  |  |  |  | | |  | | | |  |  |  |  | | | |  |  |  |
| 1 | CHILANGA |  | | | |  |  |  | |  |  |  |  |  |  |  |  |  |  | |  |  |  |
| 2 | CHILANGA |  |  |  |  |  |  |  |  |  |  |  |  |  |  |  | |  |  |  |  | | |
| 2 | CHILANGA |  |  |  |  |  |  |  |  | |  |  |  |  | | |  |  |  |  | | |  |
| 1 | LUSAKA |  |  | |  |  |  |  |  | | | | |  |  |  |  | | | |  |  |  |
| 1 | LUSAKA |  |  |  | |  |  |  |  |  |  |  |  |  |  |  |  | |  |  |  |  |  |
| 1 | LUSAKA |  |  |  |  |  |  |  |  |  | | | |  |  |  |  | | | |  |  |  |
| 1 | LUSAKA |  |  |  |  |  |  |  |  |  | | | |  |  |  |  |  | | |  |  |  |
| 1 | LUSAKA |  |  | |  |  |  |  | |  |  |  |  | | | | |  |  | | | |  |
| 1 | LUSAKA |  |  |  |  |  |  | | |  |  |  |  | | | | |  |  |  | | |  |
| 1 | LUSAKA |  |  |  | |  |  |  | | |  |  |  | | | | |  |  | |  |  |  |
| 1 | LUSAKA |  |  |  |  |  |  |  |  | |  |  |  |  |  |  |  |  |  | |  |  |  |
| 2 | LUSAKA |  |  |  |  |  |  |  |  | |  |  |  |  | | | |  |  | | |  |  |
| 2 | LUSAKA |  |  |  |  |  |  |  |  | |  |  |  |  | | | |  |  | | | |  |
| 2 | LUSAKA |  |  |  |  |  |  |  |  |  |  |  |  |  |  | |  |  |  |  |  | | |
| 2 | LUSAKA |  |  |  |  |  |  |  |  |  |  |  |  |  | |  |  |  |  |  |  | |  |
|  |  |  | | |  | | | | |  | | | | | |  |  |  |  |  |  | |  |
|  | Baseline | SKILLZ | | | Midline survey round | | | | | Endline Survey round | | | | | |  |  |  |  |  |  | |  |

Supplementary Information 1: Overview of the 12 Sessions of SKILLZ Workshops

**Overview of 12 Sessions of SKILLZ Workshops**

1// Join the SKILLZ Girl Team!

Team Identity

Team Contract

2 // I Matter!

I Matter

3 // Understanding Gender!

Defining YOU!

SKILLZ Soccer

4 // Me & My Body

Me & My Body

I Am Beautiful!

5 // Healthy Relationships

Healthy & Unhealthy Relationships

Team Handball

6 // Healthy Communication

Know Your Rights

3 SKILLZ to Say ‘NO!’

7 // Avoid Risks!

Risk Field

8 // Winning Combination

Winning Combination

9 // Rights and Responsibilities

Advocate for Yourself!

SKILLZ Soccer

10 // Build Your Team!

Find the Ball

My Supporters

11 // Let’s Get Tested!

Explain HIV Testing

Healthy Living

Supporting

12 // Goal Setting

Goal Setting

Go for Goal
