# Supplementary material for: The impact of the SKILLZ intervention on sexual and reproductive health empowerment among Zambian adolescent girls and young women: results of a cluster randomized controlled trial
Source: Reprod Health. 2025 Jun 5;22:95. doi: 10.1186/s12978-025-02046-6 (PMC12142864; doi:10.1186/s12978-025-02046-6)
Supplement: Supplementary file 1 — Supplementary Material 1. [file 12978_2025_2046_MOESM1_ESM.zip › Supplementary Table 3.docx]

| **Supplementary Table 3: Difference-in-difference effects of the SKILLZ intervention on SRE overall score and subscales among those sexually active at baseline, SKILLZ Study in Zambia** | | | | | | | | |  |
| --- | --- | --- | --- | --- | --- | --- | --- | --- | --- |
|  | **Midline^1^** | | | | **Endline^2^** | | | |  |
|  |  |  |  |  |  |  |  |  |  |
|  | D-i-D β | SE | p-value^3^ | % change | D-i-D β | SE | p-value^3^ | % change |  |
| **Overall SRE score (Range: 0-92)** ^4^ | 7.96 | 1.72 | <0.001 | 8.65% | 3.67 | 1.53 | 0.017 | 3.99% |  |
| **Subscales**^4^ |  |  |  |  |  |  |  |  |  |
| Parental support (0-16) | 1.19 | 0.583 | 0.041 | 7.00% | -0.016 | 0.47 | 0.973 | -0.09% |  |
| Comfort talking with partner (0-12) | 0.76 | 0.43 | 0.078 | 5.85% | 0.41 | 0.39 | 0.29 | 3.15% |  |
| Choice of partners, marriage, and children (0-12) | 0.66 | 0.26 | 0.013 | 5.08% | 0.30 | 0.23 | 0.2 | 2.31% |  |
| Sexual safety (0-16) | 2.11 | 0.48 | <0.001 | 12.41% | 0.45 | 0.56 | 0.42 | 2.65% |  |
| Self-love (0-16) | 0.79 | 0.32 | 0.014 | 4.65% | 0.99 | 0.25 | <0.001 | 5.82% |  |
| Sense of future (0-8) | 0.6 | 0.22 | 0.006 | 6.67% | 0.35 | 0.19 | 0.069 | 3.89% |  |
| Sexual pleasure (0-12) | 1.79 | 0.45 | <0.001 | 13.77% | 1.14 | 0.36 | 0.001 | 8.77% |  |

^1^ 399 unique participants comprising 673 participant-waves
^2^ 404 unique participants comprising 734 participant-waves
^3^ Bonferroni adjusted alpha critical value cut off: p<0.00625

^4^ Each line a separate regression model; each model adjusted for district, location, remoteness, school type, and number of students
